# Down-regulation of the brain-specific cell-adhesion molecule contactin-3 in tuberous sclerosis complex during the early postnatal period

**DOI:** 10.1186/s11689-022-09416-2

**Published:** 2022-01-15

**Authors:** Anatoly Korotkov, Mark J. Luinenburg, Alessia Romagnolo, Till S. Zimmer, Jackelien van Scheppingen, Anika Bongaarts, Diede W. M. Broekaart, Jasper J. Anink, Caroline Mijnsbergen, Floor E. Jansen, Wim van Hecke, Wim G. Spliet, Peter C. van Rijen, Martha Feucht, Johannes A. Hainfellner, Pavel Krsek, Josef Zamecnik, Peter B. Crino, Katarzyna Kotulska, Lieven Lagae, Anna C. Jansen, David J. Kwiatkowski, Sergiusz Jozwiak, Paolo Curatolo, Angelika Mühlebner, Erwin A. van Vliet, James D. Mills, Eleonora Aronica

**Affiliations:** 1 Department of (Neuro) Pathology, Amsterdam UMC, University of Amsterdam, Amsterdam Neuroscience, Amsterdam, the Netherlands; 2 Department of Neuroimmunology, Netherlands Institute for Neuroscience, Amsterdam, the Netherlands; 3 Department of Paediatric Neurology, University Medical Center, Brain Center, Utrecht, the Netherlands; 4 Department of Pathology, University Medical Center Utrecht, Utrecht, the Netherlands; 5 Rudolf Magnus Institute for Neuroscience, University Medical Center, Brain Center, Utrecht, the Netherlands; 6 Department of Pediatrics, Medical University Vienna, Vienna, Austria; 7 Institute of Neurology, Medical University Vienna, Vienna, Austria; 8 Department of Pediatric Neurology, 2nd Faculty of Medicine and Motol University Hospital, Prague, Czech Republic; 9 Department of Pathology and Molecular Medicine, 2nd Faculty of Medicine and Motol University Hospital, Prague, Czech Republic; 10 Department of Neurology, University of Maryland School of Medicine, Baltimore, MD USA; 11 Department of Neurology and Epileptology, The Children’s Memorial Health Institute, Warsaw, Poland; 12 Department of Development and Regeneration-Section Pediatric Neurology, University Hospitals KU Leuven, Leuven, Belgium; 13 Pediatric Neurology Unit, Universitair Ziekenhuis Brussel, Vrije Universiteit Brussel, Brussels, Belgium; 14 Harvard Medical School, Brigham and Women’s Hospital, Boston, MA USA; 15 Department of Child Neurology, Medical University of Warsaw, Warsaw, Poland; 16 Department of Clinical and Experimental Epilepsy, University College London, London, UK; 17 Center for Neuroscience, Swammerdam Institute for Life Sciences, University of Amsterdam, Amsterdam, the Netherlands; 18 Chalfont Centre for Epilepsy, Chalfont St Peter, UK; 19 Stichting Epilepsie Instellingen Nederland, Heemstede, the Netherlands

**Keywords:** Cerebral cortex development, Neurodevelopmental disorders, Cell adhesion, mTORopathies, Epilepsy

## Abstract

**Background:**

The genetic disorder tuberous sclerosis complex (TSC) is frequently accompanied by the development of neuropsychiatric disorders, including autism spectrum disorder and intellectual disability, with varying degrees of impairment. These co-morbidities in TSC have been linked to the structural brain abnormalities, such as cortical tubers, and recurrent epileptic seizures (in 70–80% cases). Previous transcriptomic analysis of cortical tubers revealed dysregulation of genes involved in cell adhesion in the brain, which may be associated with the neurodevelopmental deficits in TSC. In this study we aimed to investigate the expression of one of these genes – cell-adhesion molecule contactin-3.

**Methods:**

Reverse transcription quantitative polymerase chain reaction for the contactin-3 gene (*CNTN3*) was performed in resected cortical tubers from TSC patients with drug-resistant epilepsy (*n* = 35, age range: 1–48 years) and compared to autopsy-derived cortical control tissue (*n* = 27, age range: 0–44 years), as well as by western blot analysis of contactin-3 (*n* = 7 vs n = 7, age range: 0–3 years for both TSC and controls) and immunohistochemistry (*n* = 5 TSC vs *n* = 4 controls). The expression of contactin-3 was further analyzed in fetal and postnatal control tissue by western blotting and in-situ hybridization, as well as in the SH-SY5Y neuroblastoma cell line differentiation model in vitro.

**Results:**

*CNTN3* gene expression was lower in cortical tubers from patients across a wide range of ages (fold change = − 0.5, *p* < 0.001) as compared to controls. Contactin-3 protein expression was lower in the age range of 0–3 years old (fold change = − 3.8, p < 0.001) as compared to the age-matched controls. In control brain tissue, contactin-3 gene and protein expression could be detected during fetal development, peaked around birth and during infancy and declined in the adult brain. *CNTN3* expression was induced in the differentiated SH-SY5Y neuroblastoma cells in vitro (fold change = 6.2, *p* < 0.01).

**Conclusions:**

Our data show a lower expression of contactin-3 in cortical tubers of TSC patients during early postnatal period as compared to controls, which may affect normal brain development and might contribute to neuropsychiatric co-morbidities observed in patients with TSC.

**Supplementary Information:**

The online version contains supplementary material available at 10.1186/s11689-022-09416-2.

## Introduction

Tuberous sclerosis complex (TSC) is an autosomal dominant disorder, characterized by the development of benign neoplastic lesions in various organs [1]. The neurological manifestations of TSC in the brain include cortical tubers, which are malformations in the brain, representing areas of distorted cortical architecture [1–3]. TSC is associated with early onset intractable epilepsy in 60–90% of affected individuals [4], as well as a range of neuropsychiatric syndromes, collectively known as TSC-associated neuropsychiatric disorders (TAND) [5]. These include intellectual disability in about 50% of cases, autism spectrum disorder (ASD) in 40–50% cases and attention deficit hyperactivity disorder (30–40%) and is accompanied by various behavioral manifestations, such as depressed mood, anxiety and aggression, among others [4, 6, 7].

The pathogenesis of neurodevelopmental disorders has been linked to dysfunction in cell adhesion, mediated by neural cell adhesion molecules (CAMs) [8–10]. These molecules orchestrate the interactions between neurons during brain development, regulate synaptogenesis, synaptic plasticity and influence the processes of learning and memory [11, 12]. Our previous transcriptomic analysis of resected cortical tubers from patients with TSC revealed vast changes in the expression of genes associated with cell adhesion [13, 14]. This analysis highlighted a robust down-regulation of the *CNTN3* gene. Human *CNTN3* is located in the p12.3 region of the chromosome 3 and encodes for the third member of the family of six cell-adhesion molecules called contactins. Contactins belong to the superfamily of immunoglobulin-like CAMs and typically feature a glycosylphosphatidylinositol membrane anchor, four fibronectin III-like and six immunoglobulin-like domains with a significant degree of structural similarity between the family members [15]. Contactins interact with other neural CAMs, providing cues for neural cell migration, axon guidance and the organization of myelin subdomains [16]. Copy number variations (CNVs) in contactin genes are frequently observed in neurological and psychiatric disorders, including ASD, schizophrenia and intellectual disability [15, 17]. The limited evidence from rodent studies suggests that contactin-3 expression coincides with birth and it has been shown to promote neurite outgrowth in vitro [18]. Surprisingly, there is only scarce data available about contactin-3 in the human brain. Moreover, the developmental profile of contactin-3 expression in the human brain is not available, and a link with TSC has not been reported.

In this study we followed up our previous transcriptomic data and analyzed contactin-3 expression in cortical tubers in comparison to control post-mortem cortex, as well as studied its expression profile at various stages of brain development and in control brain tissue.

## Materials and methods

### Human samples

The brain tissue specimens included in this study were obtained from the archives of the department of (Neuro)Pathology of the Amsterdam University Medical Centers (Amsterdam UMC-Location AMC, the Netherlands), the University Medical Center Utrecht (UMCU, the Netherlands), Motol University Hospital (Prague, Czech Republic) and the Medical University Vienna (MUV, Austria). For the RT-qPCR analysis postnatal TSC cortical tuber samples (*n* = 35 samples, age range 0–48 years, median age 85 months) were obtained from patients who underwent resective surgery for the treatment of drug-resistant epilepsy (*n* = 30) or were autopsy-derived (*n* = 5) and postnatal control cortical samples (*n* = 27, age range 1–44 years, median age 115 months were obtained from autopsies of patients without a history of neurological diseases. For immunohistochemical analysis cortical tuber samples (n = 5, age range 2–16 years old, median age 9 years old) were analyzed in comparison to control samples (*n* = 4, age range 2–15 years old, median age 6 years old). For western blot analysis, cortical tuber samples (*n* = 7, age range of 0–3 years old, median age 24 months) and control samples (n = 7, age range 0–3 years old, median age 24 months) were used. Table 1 summarizes information about the samples used for comparison between TSC and control tissue. For temporal expression profile western blot was performed on postnatal control samples (*n* = 14, age range 1–44 years old, median age 24 months) and fetal control samples (*n* = 5, age range gestational weeks (GW) 23–41, median GW 23); and in-situ hybridization analysis was performed on control postnatal samples (*n* = 10, age range 0–67 years old, median age 54 months) and fetal control samples (*n* = 3, age range GW 14–36, median age GW 22) Fetal control samples were obtained following medically-induced abortions. Table 2 summarizes information about the samples used for the temporal expression profile in control tissue. For RNAseq analysis of 0–3 years old *n* = 4 controls, n = 10 TSC cortical tubers and *n* = 5 FCD IIB samples were used. For RNAseq analysis of 5–20 years old *n* = 6 controls, *n* = 7 TSC cortical tubers and *n* = 20 FCD IIB samples were used. These data are summarized in the **Supplementary Table** **1**. All TSC patients fulfilled the diagnostic criteria for TSC [19]. Brain tissue was either frozen and kept at − 80 °C (for molecular analysis) or fixed in 10% buffered formalin and embedded in paraffin (for histological analysis). Informed consent was obtained for the use of brain tissue and for access to medical records for research purposes. Tissue was obtained and used in accordance with the Declaration of Helsinki and the Amsterdam UMC Research Code provided by the Medical Ethics Committee, and the study was approved by the local ethical committees of all participating medical centres.

**Table 1:** Summary of sample information for comparisons between TSC and controls. Among cortical tubers used for RT-qPCR were surgery-derived (*n* = 30) and autopsy-derived (n = 5); front – frontal, temp – temporal, par – parietal cortex; WB – western blot samples; m – male, f – female

**Summary of samples for RT-qPCR (all ages)**							
**Sample**	**N**	**Median age**	**male**	**female**	**Region of brain**	**Mutation**	
**Postnatal controls**	27	85 months	12	15	front - 14, temp - 8, par - 1, cortex - 4	N/A	N/A
**Cortical tubers**	35	115 months	17	19	front - 26, temp - 9	*TSC1 - *9	*TSC2 - *26
**Samples for WB (young TSC vs control)**							
**Postnatal controls**		**Age**	**Gender**		**Region of brain**	**Mutation**	
WB CTRL1		6 weeks	m		front	–	
WB CTRL2		7 weeks	f		front	–	
WB CTRL3		4 months	m		front	–	
WB CTRL4		7 months	f		cortex	–	
WB CTRL5		1 year	f		temp	–	
WB CTRL6		2 years	f		temp	–	
WB CTRL7		3 years	m		front	–	
**Resected cortical tubers**		**Age**	**Gender**		**Region of brain**	**Mutation**	
WB TSC1		8 months	m		temp	*TSC2*	
WB TSC2		1 year	m		front	*TSC2*	
WB TSC3		2 years	m		front	*TSC2*	
WB TSC4		2 years	m		front	*TSC2*	
WB TSC5		2 years	f		front	*TSC1*	
WB TSC6		3 years	f		temp	*TSC2*	
WB TSC7		3 years	m		front	*TSC2*	
**Samples for immunohistochemistry**							
**Postnatal controls**		**Age**	**Gender**		**Region of brain**	**Mutation**	
IHC CTRL1		2 years	f		front	–	
IHC CTRL2		2 years	m		front	–	
IHC CTRL3		10 years	m		front	–	
IHC CTRL4		15 years	m		front	–	
**Resected cortical tubers**		**Age**	**Gender**		**Region of brain**	**Mutation**	
IHC TSC1		2 years	m		front	*TSC2*	
IHC TSC2		4 years	f		front	*TSC1*	
IHC TSC3		9 years	m		front	*TSC2*	
IHC TSC4		13 years	f		front	*TSC2*	
IHC TSC5		16 years	m		front	*TSC2*

**Table 2:** Summary of sample information for temporal expression profile analysis. GW – weeks of gestation, front – frontal, temp – temporal, par – parietal cortex; WB – western blot samples; m – male, f – female

**Samples for WB (temporal profile)**				
**Fetal controls**				
**N**	**Sample**	**Age**	**Gender**	**Region of brain**
1	WB FET1	22 GW	f	temp
2	WB FET2	23 GW	m	temp
3	WB FET3	23 GW	m	cortex
4	WB FET4	25 GW	m	temp
5	WB FET5	41 GW	f	front
**Postnatal controls**				
**N**	**Sample**	**Age**	**Gender**	**Region of brain**
1	WB CTRL8	1 day	m	front
2	WB CTRL9	1 month	m	front
3	WB CTRL10	3 months	f	temp
4	WB CTRL11	3 months	f	front
5	WB CTRL12	4 months	m	front
6	WB CTRL13	7 months	f	cortex
7	WB CTRL14	1 year	f	temp
8	WB CTRL15	3 years	m	front
9	WB CTRL16	2 years	f	temp
10	WB CTRL17	7 years	f	front
11	WB CTRL18	10 years	m	front
12	WB CTRL19	13 years	m	front
13	WB CTRL20	15 years	m	front
14	WB CTRL21	44 years	f	par
**Samples for in-situ hybridization (temporal profile)**				
**Fetal controls**				
**N**	**Sample**	**Age (GW)**	**Gender**	**Region of brain**
1	WB1 FET	14 GW	m	cortex
2	WB2 FET	22 GW	f	front
3	WB3 FET	36 GW	f	front
**Postnatal controls**				
**N**	**Sample**	**Age**	**Gender**	**Region of brain**
1	ISH CTRL1	1 day	m	front
2	ISH CTRL2	4 months	m	front
3	ISH CTRL3	7 months	m	front
4	ISH CTRL4	2 years	f	front
5	ISH CTRL5	2 years	m	front
6	ISH CTRL6	7 years	m	front
7	ISH CTRL7	10 years	m	front
8	ISH CTRL8	17 years	f	front
9	ISH CTRL9	44 years	m	front
10	ISH CTRL10	67 years	m	front

### Cell culture

The human SH-SY5Y neuroblastoma cell line was maintained in culture medium containing Dulbecco’s Modified Eagle Medium DMEM/F-12 (Gibco/ThermoFisher Scientific, Waltham, MA, USA), supplemented with 2 mM L-glutamine, 100 units/mL penicillin, 100 μg/mL streptomycin and 10% heat-inactivated fetal calf serum (FCS) (Gibco, Life Technologies, Grand Island, NY, USA). The cells were maintained in a 5% CO_2_ incubator at 37 °C.

For the differentiation of SH-SY5Y cells, a retinoic acid (RA) differentiation protocol was followed [20]. Briefly, the cells were plated at a density of 50,000 cells/well in 12-well plates and left for 24 h to attach. For the next 4 days, the medium content of FCS was reduced from 10% to 1% and the medium was supplemented with 10 μM all-trans-retinoic acid (RA) (Sigma-Aldrich, St. Louis, MO, USA). The cells were re-plated and cultured for another 8 days in the medium with 0% FCS, 10 μM RA, B27 supplement (Gibco/ThermoFisher Scientific, Waltham, MA, USA), 20 mM KCL, 2 mM dibutyryl-cyclic adenosine monophosphate (db-cAMP, Stemcell Technologies, Cologne, Germany) and 50 ng/ml human recombinant brain-derived neurotrophic factor (BDNF, Stemcell Technologies, Cologne, Germany). The control cells were maintained in standard medium with 1% FCS. Cells were harvested for RNA isolation 12 days after the start of serum deprivation and 8 days after the start of RA treatment.

### RNA isolation

RNA isolation from human frozen brain tissue and cell culture material was done using the miRNeasy Mini kit (Qiagen Benelux, Venlo, the Netherlands) according to the manufacturer’s instructions. The concentration and purity of RNA were determined using a Nanodrop 2000 spectrophotometer (ThermoFisher Scientific, Wilmington, DE, USA). The RNA was stored at − 80 °C until use.

### RNA-Seq library preparation and sequencing

All library preparation and sequencing were performed at GenomeScan (Leiden, the Netherlands). The NEBNext Ultra II Directional RNA Library Prep Kit for Illumina (New England Biolabs, Ipswich, MA, USA) was used for sample processing. Sample preparation was performed according to the protocol “NEBNext Ultra II Directional RNA Library prep Kit for Illumina” (NEB #E7760S/L). Briefly, mRNA was isolated from total RNA using oligo-dT magnetic beads. After fragmentation of mRNA, cDNA synthesis was performed. Next, sequencing adapters were ligated to the cDNA fragments followed by PCR amplification. Clustering and DNA-sequencing was performed using the NovaSeq6000 (Illumina, Foster City, CA, USA) in accordance with manufacturers’ guidelines. All samples underwent paired-end sequencing of 150 nucleotides in length, the mean read depth per a sample was 47 million reads.

### Bioinformatics analysis of RNA-Seq data

For the data presented in **Supplementary Fig.** **1** the final differential expression output from an RNA-sequencing (RNAseq) experiment comparing TSC cortical tubers (*n* = 12) and matched controls (*n* = 10) was provided by the data generators on request [14]. For full details on RNA-sequencing protocols used and differential expression analysis please refer to the relevant paper [14].

**Fig. 1:**
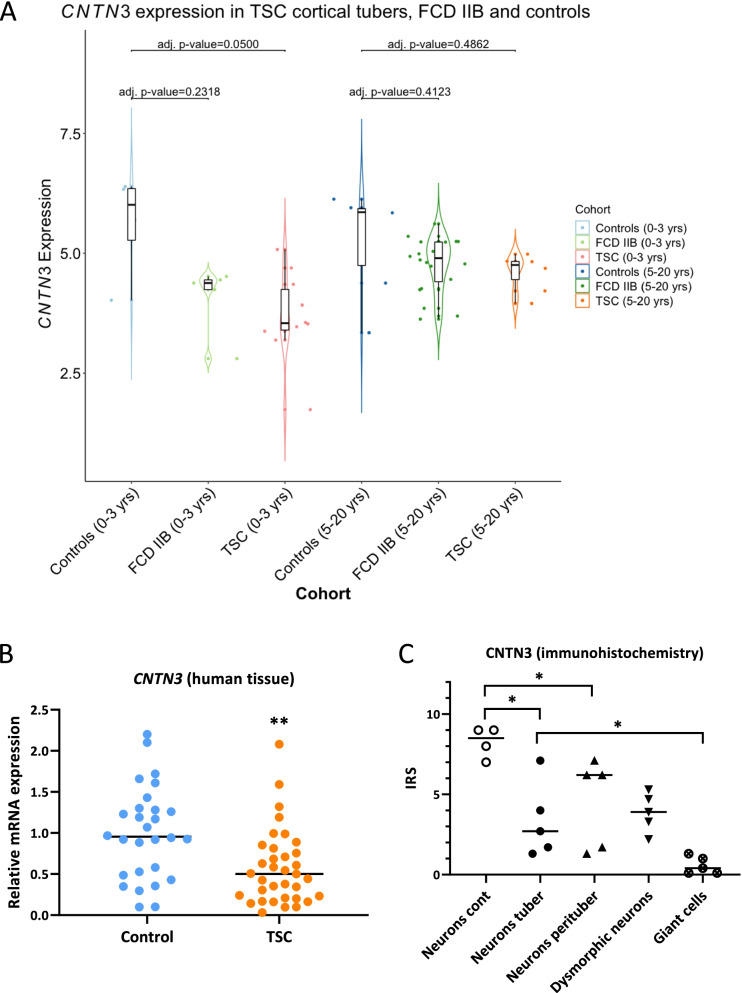
Decreased contactin-3 expression in cortical tubers. **A** – RNAseq data indicating down-regulation (adjusted *p* = 0.05) of *CNTN3* in the cohort of cortical tuber samples from 0 to 3 year old patients; **B** – RT-qPCR validation showed a down-regulation of *CNTN3* expression (fold change = − 0.5, *p* < 0.01) in cortical tubers (*n* = 35) compared to autopsy-derived cortical control tissue (*n* = 27); median, **p < 0.01, Mann-Whitney U test; **C** – Semiquantitative analysis of contactin-3 immunoreactivity, showing lower neuronal IRS in cortical tubers and in perituberal areas (*n* = 5) compared to neurons in control cortex (*n* = 4); *p < 0.01, Student’s t-test

For the data presented in Fig. 1 the following procedure was used. The Bestus Bioinformaticus Decontamination Using Kmers (BBDuk) tool from the BBTools suite was used for adapter removal, quality trimming and removal of contaminant sequences (ribosomal/bacterial) [21]. A phred33 score of 20 was used to assess the quality of the read, any read shorter than 31 nucleotides in length was excluded from the down-stream analysis.

Reads were aligned directly to the human GRCh38 reference transcriptome (Gencode version 33) [22] using Salmon v0.11.3 [23]. Transcript counts were summarized to the gene level and scaled used library size and average transcript length using the R package tximport [24]. Genes detected in less than 20% of the samples in any diagnosis and with counts less than 6 across all samples were filtered out. The gene counts were than normalized using the weighted trimmed mean of M-values (TMM) method using the R package edgeR [25]. The normalized counts were than log2 transformed using the voom function from the R package limma [26]. The subsequent differential expression was carried out using the R package limma. Subsequently, a linear model was fit for each gene and moderated t-statistic was calculated after applying an empirical Bayes smoothing to the standard errors. Those genes with a Benjamini-Hochberg adjusted *p*-value < 0.05 were considered differentially expressed. Please refer to **Supplementary Table** **1** for more information about samples.

### Reverse transcription (RT) and quantitative polymerase chain reaction (qPCR)

Total RNA (2000 ng for brain tissue or 250 ng for cell culture material) was reverse-transcribed using oligo-dT primers in 25 μL of mix. The cDNA was further diluted with RNase-free water (10 times for brain tissue or 3 times for cell culture material) and stored at − 20 °C until use.

To evaluate mRNA expression each qPCR reaction contained 1 μl cDNA, 2.5 μl of FastStart Reaction Mix SYBR Green I (Roche Applied Science, Indianapolis, IN, USA) and 0.4 μM of both reverse and forward primers. The final volume was adjusted to 5 μl with RNase-free water. The cycling conditions were carried out as follows: initial denaturation at 95 °C for 5 min, followed by 45 cycles of denaturation at 95 °C for 15 s, annealing at 65 °C for 5 s and extension at 72 °C for 10 s. The fluorescent product was measured by a single acquisition mode at 72 °C after each cycle. The primers used for the qPCR were: contactin-3 (*CNTN3*, forward: 5′-GAGGGGATGGGACCAGTAGT-3′; reverse: 5′-GTGGACATTCGAGATGGCTGA-3′), elongation factor 1 alpha (*EEF1A1*, forward: 5′-ATCCACCTTTGGGTCGCTTT-3′; reverse: 5′-CCGCAACTGTCTGTCTCATATCAC-3′), glyceraldehyde 3-phosphate dehydrogenase (*GAPDH*, forward: 5′- AGGCAACTAGGATGGTGTGG-3′; reverse: 5′- TTGATTTTGGAGGGATCTCG-3′), chromosome 1 open reading frame 43 (*C1orf43*, forward: GATTTCCCTGGGTTTCCAGT; reverse: ATTCGACTCTCCAGGGTTCA). The geometric mean of *EEF1A1* and *GAPDH* expression was used for the normalization of RT-qPCR. Normalization of data in SH-SY5Y differentiation experiment was done relative to the *C1orf43* expression. Quantification of data was performed using LinRegPCR software [27] as previously described [28].

### Protein isolation and western blot analysis

Protein extraction from human brain tissue was done by homogenization of tissue in lysis buffer (50 mM Tris-HCl pH 7.4, 150 mM of NaCl, 1% NP-40, 0.5% sodium deoxycholate) supplemented with the mixture of EDTA-free protease and phosphatase inhibitors (Roche Diagnostics, Almere, the Netherlands). The homogenates were centrifuged at 12,000 x g for 10 min and the supernatant was used for further analysis. Protein content was determined using the bicinchoninic acid method.

Equal amounts of protein (20 μg/lane) were separated using sodium dodecyl sulfate polyacrylamide gel electrophoresis on a gradient Bolt 4–12% Bis-Tris Plus gel (ThermoFisher Scientific, Wilmington, DE, USA). Subsequently, separated proteins were transferred onto polyvinylidene difluoride membranes (Immobilon-P; Merck, Darmstadt, Germany) for 90 min at 100 V, using a wet electroblotting system (BioRad, Hercules, CA, USA). The blots were blocked for 1 h in 5% non-fat dry milk in Tris-buffered saline-Tween20 (TBS-T; 20 mM Tris, 150 mM NaCl, 0.1% Tween 20, pH 7.5). Blots were incubated overnight at 4 °C with primary antibodies: anti-contactin-3 (1:200, goat polyclonal, AF5539, R&D Systems, Minneapolis, United States) and anti-β-actin (1:30,000, mouse monoclonal, clone C4, AB, Merck, Darmstadt, Germany). After several washes in in TBS-T, blots were incubated with secondary antibodies donkey anti-goat immunoglobulin-HRP (1:2500, Rockland Immunochemicals, Limerick, PA, USA) or goat anti-mouse immunoglobulin-HRP (1:2500, Dako, Glostrup, Denmark) for 1 h. After several washes in TBS-T, immunoreactivity was visualized using ECL PLUS Western blotting detection reagent (GE Healthcare Europe, Diegen, Belgium). The expression of β-actin was used as loading control. Chemiluminescent signal was detected using an ImageQuant LAS 4000 analyzer (GE Healthcare, Eindhoven, the Netherlands). A Precision Plus Protein Dual Color Standard (Bio-Rad, Richmond, CA, USA) was used to determine the molecular weight of the proteins. For the quantitative analysis of the blots, the band intensities were measured densitometrically using ImageJ software (U.S. National Institutes of Health, Bethesda, MD, USA).

### Immunohistochemistry

Immunohistochemistry was performed on 6 μm-thick FFPE tissue. The sections were deparaffinized in xylene, rinsed in ethanol (100%, 95%, 70%), and incubated in 0.3% hydrogen peroxide in methanol for 20 min. Antigen retrieval was performed using a pressure cooker in 0.1 M citrate buffer, pH 6.0 at 120 °C for 10 min. Slides were washed with phosphate-buffered saline (PBS; 0.1 M, pH 7.4) and incubated overnight with the primary antibody in normal antibody diluent (Klinipath, Olen, Belgium) at 4 °C. The primary antibodies used were: anti-contactin-3 (1:300 rabbit polyclonal ab203592, Abcam, Cambridge, UK), anti-synaptophysin (1:500 mouse monoclonal, DAK-SYNAP, Dako, Glostrup, Denmark). After that, the sections were washed in PBS and incubated with the corresponding secondary antibodies using a polymer-based peroxidase immunocytochemistry detection kit (Brightvision plus kit, ImmunoLogic, Duiven, the Netherlands). After washing, the sections were stained with 3,3′-diaminobenzidine tetrahydrochloride (0.5 mg/ml DAB, Sigma-Aldrich, St. Louis, MO, USA) in the presence of hydrogen peroxide in Tris-HCl buffer (50 mM, pH 7.6). The sections were counterstained with hematoxylin, dehydrated in alcohol and xylene, and coverslipped.

For immunohistochemistry with double-labelling slides were incubated with the primary antibody against contactin-3, mouse anti-glial fibrillary acidic protein (GFAP; 1:4000, Sigma-Aldrich, St. Louis, MO, USA), mouse anti-NeuN (1:2000, MAB377, Chemicon, Temecula, CA, USA) and mouse anti-human leukocyte antigen (HLA-DR/DP/DQ; 1:100, clone CR3/43 Agilent, Santa Clara, CA, USA). The slides were then incubated with the alkaline phosphatase (AP)-conjugated secondary antibodies (anti-rabbit-AP, DPVR55AP, Brightvision plus kit, ImmunoLogic, Duiven, The Netherlands) for 30 min. The chromogenic reaction was developed with Vector Blue (#SK-5300; Vector Labs), which produced blue signal. Next, secondary horseradish peroxidase (HRP)-conjugated antibodies (anti-mouse-HRP, DPVR55HRP, Brightvision plus kit, ImmunoLogic, Duiven, The Netherlands) were applied for 30 min. The chromogenic reaction was developed with 3-amino-9-ethylcarbazole (AEC; Sigma-Aldrich, St. Louis, MO, USA), producing red signal.

Quantitative evaluation of immunoreactivity was performed using immunoreactivity score (IRS) approach. Semiquantitative evaluation of immunoreactivity was performed for contactin-3 and synaptophysin immunostaining using an Olympus microscope and examining each section with high-power nonoverlapping fields (of 0.0655 mm × 0.0655 mm width; each corresponding to 4.290 μm^2^; using a square grid inserted into the eyepiece). The staining intensity of the immunoreactive signal was evaluated in neurons, dysmorphic neurons and giant cells using a scale of 1–4 (1: no; 2: weak; 3: moderate; 4: strong signal). This score represents the predominant staining intensity found as averaged from the selected fields. Furthermore, the relative number of positive cells (0: no; 1: single to 10%; 2:11–50%; 3: > 50%) was also evaluated in these areas. Then the IRS was calculated by multiplying the intensity score by the relative number score.

### In-situ hybridization

FFPE brain tissue was deparaffinized in xylene and rinsed in ethanol (2X 100%, 1X 70%) and sterile water. Antigen retrieval was performed using a pressure cooker in sodium citrate buffer, pH 6.0, at 121 °C for 10 min. The tissue sections were incubated with the probe against human *CNTN3* (/5DigN/+TmUmU+CmCmC+AmCmU+GmUmG+TmUmU+CmAmG+C/3Dig_N/, where DigN indicates digoxigenin labels, m indicates a 2-O-methyl modification and + indicates a locked nucleic acid (LNA) modification) at 100 nM in hybridization mix (600 mM NaCl, 10 mM HEPES, 1 mM EDTA, 5X Denhardts, 50% formamide) for 1 h at 60 °C. Sections were washed with 2X saline-sodium citrate buffer (SSC) for 2 min, 0.5X SSC for 2 min and 0.2X SSC for 1 min (in agitation). After washing with sterile PBS, sections were blocked for 15 min with 1% bovine serum albumin (BSA), 0.02% Tween 20 and 1% normal goat serum. Hybridization was detected with sheep alkaline phosphatase (AP)-labeled anti-DIG antibody (1:1500, Roche Applied Science, Basel, Switzerland). Nitro-blue tetrazolium chloride (NBT)/5-bromo-4-chloro-3′-indolyphosphate p-toluidine salt (BCIP) was used as chromogenic substrate for alkaline-phosphatase detection. NBT/BCIP was diluted 1:50 in NTM-T buffer (100 mM Tris, pH 9.5; 100 mM NaCl; 50 mM MgCl_2_; 0.05% Tween 20). Negative control assays were performed without probes (sections were blank).

### Statistical analysis

Statistical analyses were performed using Graphpad prism 5 (Graphpad Software, San Diego, CA, USA). The Mann-Whitney U-test or Student’s t-test were used for comparisons between groups. A value of *p* < 0.05 was assumed to indicate a significant difference.

## Results

### Contactin-3 expression is down-regulated in cortical tubers

RNAseq analysis of the contactin-3 family genes revealed *CNTN3* as the only significantly down-regulated gene of the contactin family in cortical tubers (log_2_ fold change = − 1.32, q-value = 0.004, *n* = 12 TSC vs *n* = 10 controls) compared to the autopsy-derived control tissue (**Supplementary Fig.** **1**). Further analysis of RNAseq data showed that *CNTN3* was down-regulated (adjusted *p*-value = 0.05) in the cortical tuber samples obtained from patients of 0–3 years old and a trend for lower *CNTN3* was also observed in the 0–3 years group of samples from the patients with focal cortical dysplasia (FCD) type IIB compared to age-matched controls (Fig. 1A). However, no difference was observed between TSC or FCDIIB and age-matched controls at the ages 5–20 years old. RT-qPCR analysis in a larger TSC cohort (n cortical tubers = 35 vs n controls = 27) also showed a decreased expression of *CNTN3* (fold change = − 0.5, *p* < 0.001; Fig. 1B).

The analysis of contactin-3-immunoreactive cells in the frontal cortex showed that the IRS (see materials and methods) was lower in neurons (*p* < 0.05) in cortical tubers (*n* = 5 samples) compared to controls (*n* = 4 samples), as well as in the perituberal areas (p < 0.05; Fig. 1C). Interestingly, the lowest IRS value was observed in the youngest 2 TSC cases (2 and 4 years old). There was no difference in IRS between neurons in the cortical tubers, in the perituberal areas and dysmorphic neurons (Fig. 1C). The intensity of contactin-3 immunoreactivity was also reduced (p < 0.05) in tuber neuropil compared to control (**Supplementary Fig.** **1****B**), which was accompanied with lower immunoreactivity for synaptophysin (**Supplementary Fig.** **1****C**).

**Fig. 2:**
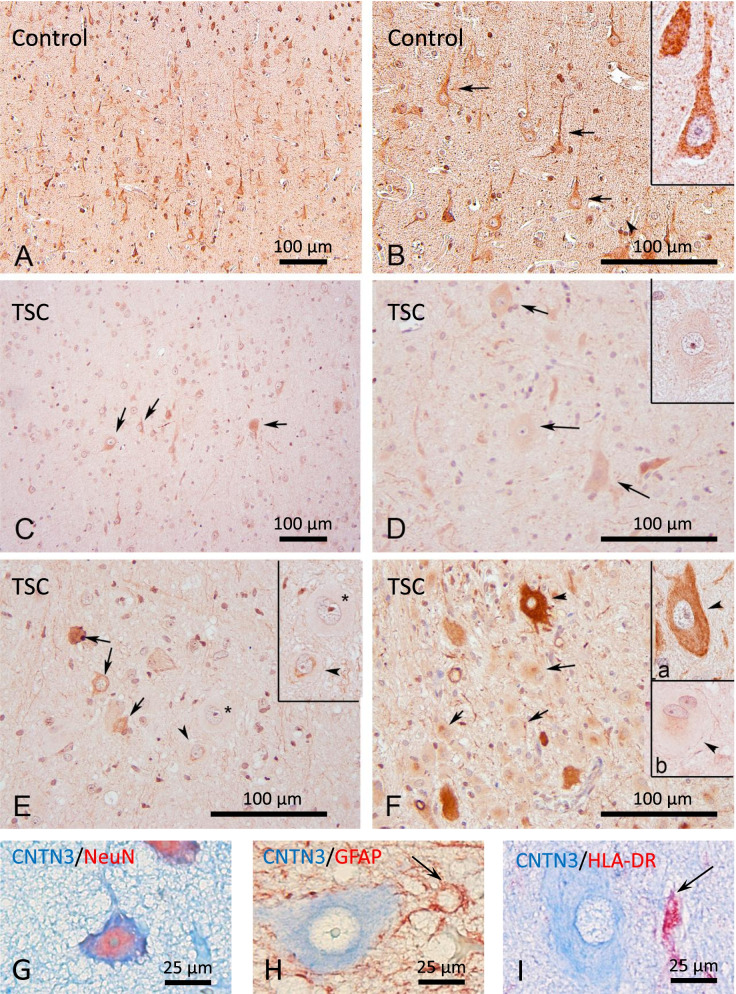
Immunohistochemistry for contactin-3. **A** – contactin-3 expression was observed in neuronal soma/neuropil in the autopsy-derived control cortex; **B** – arrows and inset indicate contactin-3-immunoreactive pyramidal neurons; **C** – weaker staining and more sparsely located contactin-3-immunoreactive neurons were observed in cortical tubers (arrows); **D** – balloon cells were mostly contactin-3-negative or showed perinuclear expression (**E**, arrows, inset); **F** – some dysmorphic neurons showed strong contactin-3 immunoreactivity (arrowhead in **F** and inset **a**); scale bar 100 μm; (**G-I**) – double-labelling of contactin-3 (blue) with cell-type specific markers, NeuN for neurons (**G**), GFAP for astrocytes (**H**) and HLA-DR for microglia, scale bar – 25 μm

Immunohistochemistry showed a predominantly neuronal expression of contactin-3 in control tissue (Fig. 2A), with strong immunoreactivity observed in pyramidal neurons (Fig. 2B). Variable contactin-3 immunoreactivity was found in cortical tubers with regions of less staining and sparsely located cells with strong expression (Fig. 2C). Based on the morphology of dysmorphic cells observed in the tubers, it was found that balloon cells showed only weak contactin-3 immunoreactivity in the cytoplasm (Fig. 2D**; F inset b**) or in the perinuclear regions (Fig. 2E), whilst the strongest immunoreactivity was observed in some dysmorphic neurons (Fig. 2F**; F inset a**). Consistent with neuronal expression reported in literature, double-labelling of contactin-3 with cell-type specific markers demonstrated that it was strongly expressed in neurons (Fig. 1G), whilst virtually absent in astrocytes (Fig. 1H) and microglia (Fig. 1I).

### Reduced expression of contactin-3 during early postnatal period

**Fig. 3:**
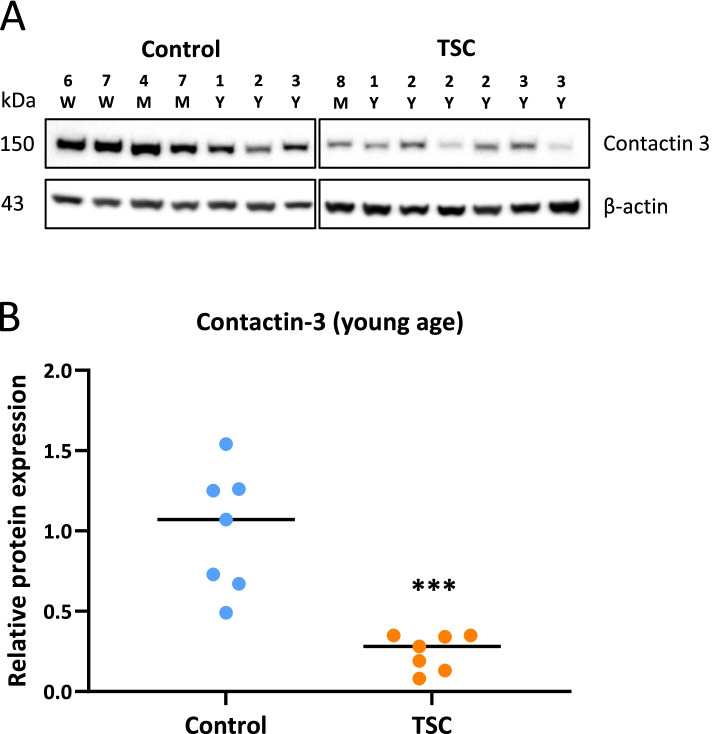
Decreased contactin-3 expression in cortical tubers during early postnatal period. **A** – western blot for contactin-3 in autopsy-derived control samples (age range: 6 weeks-3 years) and cortical tuber samples (age range: 8 months-3 years); **B** – optical density analysis showed a lower (fold change = − 3.8, *p* < 0.001) contactin-3 expression in cortical tubers (*n* = 7) compared to controls (n = 7); W – weeks, M – months, Y – years of age; median, ***p < 0.001, Mann-Whitney U test

We further assessed contactin-3 expression in the group of samples from young TSC patients (age range: 0–3 years old, *n* = 7) compared to age-matched controls (age range: 0–3 years old, n = 7). Western blot analysis showed lower expression in cortical tubers (Fig. 3A, B) as compared to control cortex (fold change = − 3.8, *p* < 0.001).

**Fig. 4:**
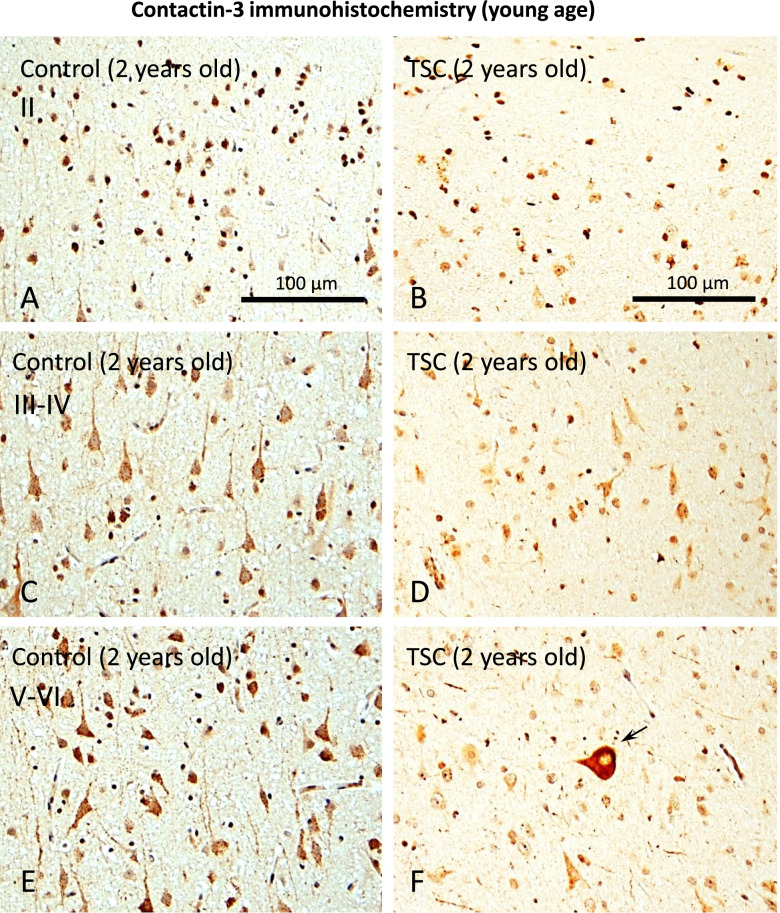
Immunohistochemistry for contactin-3 (young age). Contactin-3 expression was analyzed in a resected cortical tuber sample from a 2-year-old patient and compared to the 2-year-old autopsy-derived control; **A, B** – expression in the layer II of cerebral cortex; **C, D** – expression in the layers III-IV; **E, F** – expression in the layers V-VI; overall, the observed immunoreactivity was reduced in cortical tubers. In contrast, single dysmorphic neurons (arrow in **F**) showed strong immunoreactivity; scale bar 100 μm

Immunohistochemical analysis of the cortical tubers from young patients showed an overall weaker neuronal expression of contactin-3 in the tuber compared to control throughout the frontal cerebral cortex (layer II: Fig. 4; **A, B** layers III-IV: **C, D**; layers V-VI: **E, F**; the tuber and control samples from 2-year-old patients are represented). It was observed that contactin-3 immunoreactivity was weak in individual neurons in the cortical tubers, however, strong immunoreactivity could be seen in dysmorphic neurons (Fig. 4F, arrow).

### Contactin-3 expression is developmentally regulated with a peak expression during early postnatal period

**Fig. 5:**
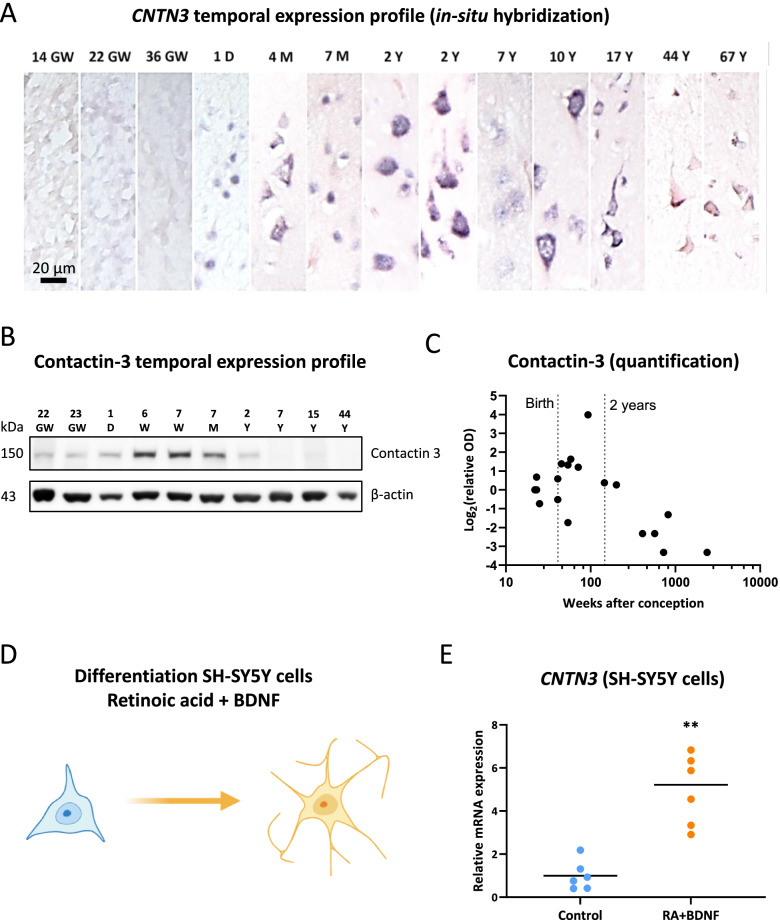
Contactin-3 is developmentally regulated in the human brain. **A** – In-situ hybridization for *CNTN3* in autopsy-derived fetal and postnatal control cerebral cortex (age range: gestational week 14–67 years); **B** – western blot for contactin-3 in the control cortex (age range: gestational week (GW) 22–44 years); **C** – optical density analysis of contactin-3 expression across different ages; **D** – schematic representation of the SH-SY5Y cell differentiation; **E** – RT-qPCR showed increased expression of *CNTN3* expression in differentiated SH-SY5Y cells (fold change = 6.2, p < 0.01, *n* = 6); GW – weeks of gestation, W – weeks, D – days, M – months, Y – years of age; RA – retinoic acid; scale bar 20 μm; **p < 0.01, Mann-Whitney U test

We further analyzed the temporal profile of contactin-3 expression in the human control autopsy-derived frontal cortex (Fig. 5A). In-situ hybridization using a *CNTN3*-specific probe showed weak hybridization signal in the fetal brain at the ages of 14–36 weeks of gestation (GW); stronger in-situ hybridization signal appeared in neurons in the postnatal samples as early as 1 day of age and was present in the samples from infancy to at least 17 years of age; weaker hybridization signal was observed in the adult (44 years old) and aged brain (67 years old; Fig. 5A). Western blot analysis showed a similar pattern of contactin-3 immunoreactivity with the strongest band observed in the samples from patients several months of age, however immunoreactivity was barely detectable in the adult brain (Fig. 5B). Quantification of the relative optical density (OD) showed that the highest protein expression could be observed around birth and during infancy, with the expression declining in later ages (Fig. 5C).

We further hypothesized that the expression of contactin-3 could depend on the differentiation state of neurons during brain development. Therefore, we used the SH-SY5Y neuroblastoma cell line, in which neuronal differentiation was induced with retinoic acid and brain-derived neurotrophic factor (BDNF). Over time these cells developed neurites (Fig. 5D). RT-qPCR analysis showed indeed that *CNTN3* expression was induced (fold change = 6.2, *p* < 0.01, *n* = 6) in the differentiated SH-SY5Y cells (Fig. 5E).

## Discussion

We investigated the expression of a CAM contactin-3 in the cortical tubers resected from patients with TSC and compared these to control post-mortem cortical tissue. Our results indicate that contactin-3 is down-regulated in cortical tubers on RNA and protein levels, especially during the early postnatal period. The peak of contactin-3 expression in the control cortex could be observed during infancy, with a marked decrease in the adult brain. Additionally, *CNTN3* expression was induced in neuronal cells during differentiation in vitro.

The members of the contactin family are enriched in the brain and participate in various processes of brain development, such as proliferation, differentiation, and migration of neural cells, axon guidance, as well as formation and organization of synapses [29, 30]. The dysregulation of these processes is known to contribute to neurodevelopmental disorders. In humans three of the contactin family genes, *CNTN3*, *CNTN4* and *CNTN6* are located on the short arm of the chromosome 3. Deletions in this region frequently result in the 3p-deletion syndrome – a genetic disorder associated with ASD, schizophrenia, epilepsy and accompanied by developmental delay and intellectual disability [10]. Copy number variations in 3p-deletion syndrome often occur at the 3p26 locus (which contains *CNTN4*, *CNTN6*) and one case report of a child with a proximal interstitial 3p deletion that included *CNTN3* (located at 3p12) also demonstrated neurodevelopmental delay, growth retardation and dysmorphic facial features among other abnormalities [31]. Deleterious variants and polymorphisms in contactin genes have also been implicated in ASD, including *CNTN3* [32, 33], *CNTN4* [34–36], *CNTN5* [37], *CNTN6* [38], as well as contactin-associated protein 2 (*CNTNAP2*) [39]. The abovementioned neuropathological features often present in TSC, which is characterized by a range of neuropsychiatric disorders, encompassed by TAND [40]. Our study showed a significant down-regulation of contactin-3 expression in TSC cortical tubers, which was not caused by a deletion or a specific mutation in *CNTN3*. A similar trend could be observed in FCD IIB – another mechanistic target of rapamycin (mTOR)-related malformation of cortical development [41]. The causes for the overall decrease in contactin-3 might include reduced expression in individual normal-appearing neurons and dysmorphic neurons, as well as decreased total density of neurons and disordered cellular architecture in the cortical tubers. Moreover, the effect of the pathology on contactin-3 seems to affect not only tubers, but also the surrounding perituberal areas, which may compromise its function on a larger scale. In addition, contactin-3 is itself a synaptic protein and its insufficiency may affect regions distant from the tubers. Therefore, the decrease in contactin-3 might be an indirect result of mTOR deregulation and contribute to the complex pathophysiology of TSC and related disorders.

The emergence of contactin-3 in the brain around birth and peak of expression in the infant period suggest a role for contactin-3 in late corticogenesis. The disturbances in contactin-3 expression might contribute to the development of neuropsychiatric phenotype in TSC. In humans, contactin-3 (previously known as PANG/BIG-1) has been shown to be specifically expressed in the brain with the highest levels in the cerebral cortex, cerebellum and amygdala [42]. *CNTN3* transcripts could be detected in the rat brain shortly after birth, at postnatal day 2, and reach its maximum in the adult brain [18]. In accordance with this evidence, we found that in the human cortex, strong contactin-3 expression could be observed around birth and during early postnatal period. However, we also detected weak contactin-3 expression in fetal cortical tissue. This difference could be explained by either a specific human trait or a higher method sensitivity of our analysis. The down-regulation of contactin-3 in the cortical tubers occurs during the expected peak of its protein expression in infancy, before the maximal synaptic density is reached in the human frontal cortex [43]. Therefore, it is possible that a lack of contactin-3 during this period impairs its functions, most important in a narrow window of early postnatal development, but not later in life. Such functions include the neurite outgrowth-promoting activity previously shown for contactin-3 in vitro [18]. Accordingly, *CNTN3* expression can be induced in a neuronal cell line during differentiation, which is accompanied by the extension of neurites. This suggests a functional connection between contactin-3 and neuronal maturation. The lack of contactin-3 in cortical tubers and perituberal areas may therefore affect this process and result in insufficient or aberrant synaptic wiring of cortical neurons after birth, contributing to the development of neuropsychiatric and cognitive abnormalities observed in children with TSC, such as ASD, neurodevelopmental delay and intellectual disability.

However, the observed decrease in contactin-3 may indicate only a delay in brain development in affected regions, which could be a temporary phenotype, characteristic of early years of life, but with potentially profound effects on brain development. Cortical tuber formation is a complex and dynamic process characterized by the development of (sub) cortical lesions during the early stages of corticogenesis [44–46] and further evolving over time [47, 48]. The observation that contactin-3 in tubers does not differ in expression with control as the age of subjects advances, supports this notion. However, the period when it has to be highly expressed is the infancy, and its time-dependent physiological function might be missing in TSC.

Particularly interesting in the context of TSC is the structural similarity of contactin-3 to other immunoglonulin-like CAMs**.** It displays up to 60% homology with other contactins [49] and 19–30% homology with other immunoglobulin-like molecules, including neural cell adhesion molecule (NCAM) and L1 family CAMs [18]. Such structural similarity may confer overlapping and redundant functions on these CAMs. It has been observed that larger copy number variations, affecting multiple genes in 3p-deletion syndrome, show a higher penetrance with more severe phenotypes [10]. Based on our RNAseq data, down-regulation in *CNTN1*, *CNTN4* and *CNTN5* is also present in cortical tubers to some degree, albeit to a lesser extent than *CNTN3*. Dysregulation of multiple contactins and other CAMs simultaneously in cortical tubers may amplify the pathological phenotypes observed in TSC. Reduced expression of contactin-3 is likely to be one element in a complex network of interactions impairing neural development. Such interaction network may involve immunoglobulin-like molecules or receptors, especially located on the chromosome 3p. One of the potential interacting partners is the protein tyrosine receptor phosphatase type G (PTPRG), a neuronal receptor that has been shown to bind contactin-3 in vitro [50] and in vivo at the surface of photoreceptor cells in the mouse retina [51]. In order to better understand the basis for the development of neuropsychiatric disorders in TSC, the future analysis should be more network-oriented and include CAMs like contactin-3 and their functionally and structurally associated partners.

## Conclusions

Our results indicate that contactin-3 is developmentally regulated in the human brain and expressed most highly during the early postnatal period. Contactin-3 is down-regulated in cortical tubers during the the first months to years posnatally – a critical window for brain development. The expression of contactin-3 in the normal brain is estimated to peak during this period and is likely to be involved in maturation of neurons. Therefore, the lack of contactin-3 might contribute to aberrant corticogenesis and development of neuropsychiatric manifestations in patients with TSC. Further analysis is warranted for contactin-3 and the associated network of proteins in TSC as well as other neurodevelopmental disorders.

## Supplementary Information

**Additional file 1.** .

**Additional file 2.**

## Abbreviations

ASD: Autism spectrum disorders
BDNF: Brain-derived neurotrophic factor
CAM: Cell adhesion molecules
CNTN: Contactin
CNV: Copy number variations
GW: Gestational week
FFPE: Formalin-fixed paraffin-embedded
IHC: Immunohistochemistry
OD: Optical density
qPCR: Quantitative polymerase chain reaction
RA: Retinoic acid
RT: Reverse transcription
TAND: TSC-associated neuropsychiatric disorders
TSC: Tuberous sclerosis complex
WB: Western blot

## Declarations

### Availability of data and materials

The data that supports the findings of this study are available from the corresponding author upon reasonable request.
