# Supplementary material for: Down-regulation of the brain-specific cell-adhesion molecule contactin-3 in tuberous sclerosis complex during the early postnatal period
Source: J Neurodev Disord. 2022 Jan 15;14:8. doi: 10.1186/s11689-022-09416-2 (PMC8903535; doi:10.1186/s11689-022-09416-2)
Supplement: Supplementary file 1 — Additional file 1. . [file 11689_2022_9416_MOESM1_ESM.docx]

**Supplementary Table 1: Clinical information of TSC, FCD IIB and autopsy control cases (0-3 yo)**

| **ID** | **PA Diagnosis** | **Gender** | **Onset age (y)** | **Age (during operation)** | **Seizure frequency (month)** | **Seizure type** | **Area of resection** | **AED** | **Mutation** |
| --- | --- | --- | --- | --- | --- | --- | --- | --- | --- |
| C16 ^a^ | Control | f | NA | 0 | NA | NA | C | NA | NA |
| C18 ^a^ | Control | f | NA | 0 | NA | NA | C | NA | NA |
| C19 ^a^ | Control | f | NA | 2 | NA | NA | C | NA | NA |
| C22 ^a^ | Control | f | NA | 0 | NA | NA | C | NA | NA |
| F11 ^a^ | FCD 2b | m | 1 | 3 | 152 | Focal | P | CLB, OXC | NA |
| F31 ^a^ | FCD 2b | m | 0 | 2 | 30 | Focal | F | PHT, TPM, VPA, VGB | NA |
| F36 ^a^ | FCD 2b | m | 0 | 3 | 30 | Focal | F | CBZ, LCS | NA |
| F41 ^a^ | FCD 2b | f | 0 | 3 | 517 | Focal | F | CBZ, CLB, LEV, OXC, VPA | MTOR |
| F66 ^a^ | FCD 2b | m | 1 | 2 | 91 | Focal | F | CBZ | NA |
| T2 ^a^ | TSC | m | 0 | 2 | 122 | Focal | F | LEV, VGB | TSC2 |
| T12 ^a^ | TSC | f | 0 | 2 | 304 | Focal | F | CNP, LEV, OXC, ZNS | TSC1 |
| T13 ^a^ | TSC | m | 0 | 2 | 152 | Focal | F | ZNS | TSC2 |
| T14 ^a^ | TSC | m | 0 | 2 | 304 | Focal | F | CLB, LEV, PB, VGB | TSC2 |
| T15 ^a^ | TSC | m | 0 | 1 | 243 | Focal | F | CLB, VGB, ZNS | TSC2 |
| T16 ^a^ | TSC | m | 0 | 0 | 61 | Focal | F | CLB, LEV, VGB | TSC2 |
| T18 ^a^ | TSC | m | 0 | 0 | 304 | Focal | F | CBZ, LEV, VGB | TSC2 |
| T19 ^a^ | TSC | m | 2 | 3 | 457 | Focal | F | LEV | TSC1 |
| T22 ^a^ | TSC | v | 0 | 0 | 152 | Focal | F | CLB, LEV, VGB | TSC2 |
| T24 ^a^ | TSC | f | 0 | 0 | 152 | Focal | F | LEV, OXC, VGB | TSC2 |

m = male, f = female, y = year, C = cortex, F = frontal, T = temporal, P = parietal, O = occipital, CBZ = Carbamazepine, CLB = Clobazam, CNP = Clonazepam, LCS = Lacosamide, LEV = Levetiracetam, LMT = Lamotrigine, OXC = Oxcarbazepine, PB = Phenobarbital, PER = Perampanel, PHT = Phenytoin, TPM = Topiramate, VGB = Vigabatrin, VPA = Valproic.acid, ZNS = Zonisamide, ^a^ = RNA sequencing, ^b^ = IHC validation, ^c^= Functional Experiments

**Supplementary Table 1: Clinical information of TSC, FCD IIB and autopsy control cases (5-20 yo)**

| **ID** | **PA Diagnosis** | **Gender** | **Onset age (y)** | **Age (during operation)** | **Seizure frequency (month)** | **Seizure type** | **Area of resection** | **AED** | **Mutation** |
| --- | --- | --- | --- | --- | --- | --- | --- | --- | --- |
| C17 ^a^ | Control | m | NA | 10 | NA | NA | C | NA | NA |
| C20 ^a^ | Control | m | NA | 15 | NA | NA | C | NA | NA |
| C21 ^a^ | Control | m | NA | 13 | NA | NA | C | NA | NA |
| C23 ^a^ | Control | m | NA | 10 | NA | NA | C | NA | NA |
| C24 ^a^ | Control | f | NA | 17 | NA | NA | C | NA | NA |
| C25 ^a^ | Control | f | NA | 17 | NA | NA | C | NA | NA |
| F4 ^a^ | FCD 2b | f | 3 | 9 | 9 | Focal | F | OXC | MTOR |
| F5 ^a^ | FCD 2b | m | 1 | 14 | 457 | Focal | F | LMT, PHT, VPA | MTOR |
| F6 ^a^ | FCD 2b | m | 3 | 5 | 152 | Focal | F | PHT, VPA, VGB | MTOR |
| F7 ^a^ | FCD 2b | m | 4 | 18 | 91 | Focal | F | CLB, OXC, VPA | NA |
| F8 ^a^ | FCD 2b | m | 2 | 18 | 91 | Focal | F | CBZ, LCS, LMT, VPA | MTOR |
| F9 ^a^ | FCD 2b | m | 12 | 17 | 1 | Focal | F | LMT, OXC | MTOR |
| F10 ^a^ | FCD 2b | m | 3 | 5 | 304 | Focal | O | CBZ, CLB | NA |
| F12 ^a^ | FCD 2b | f | 4 | 10 | 91 | Focal | F | OXC, VPA | TSC1 |
| F15 ^a^ | FCD 2b | f | 0 | 11 | 17 | Focal | F | LMT, OXC | NA |
| F16 ^a^ | FCD 2b | m | 0 | 6 | 609 | Focal | F | VPA | NA |
| F18 ^a^ | FCD 2b | m | 15 | 18 | 91 | Focal | F | CNP, LEV, OXC | NA |
| F21 ^a^ | FCD 2b | m | 3 | 8 | 913 | Focal | P | CBZ, LMT, VPA | MTOR |
| F26 ^a^ | FCD 2b | f | 3 | 5 | 122 | Focal | F | OXC, TPM, VGB | NA |
| F28 ^a^ | FCD 2b | f | 1 | 9 | 30 | Focal | F | OXC | NA |
| F35 ^a^ | FCD 2b | f | 1 | 8 | 30 | Focal | F | CBZ, PER, PHT | NA |
| F37 ^a^ | FCD 2b | f | 10 | 10 | 30 | Focal | F | CLB, TPM | NA |
| F40 ^a^ | FCD 2b | f | 3 | 8 | 609 | Focal | O | CBZ, CLB, LEV, OXC, VPA | NA |
| F59 ^a^ | FCD 2b | m | 3 | 10 | 30 | Focal | F | CBZ, VPA | MTOR |
| F60 ^a^ | FCD 2b | f | 8 | 18 | 61 | Focal | F | CBZ, CLB, LMT, TPM, VPA | MTOR |
| F61 ^a^ | FCD 2b | f | 2 | 17 | 1217 | Focal | P | CLB, LCS, LMT, PER, VGB | NA |
| T1 ^a^ | TSC | f | 0 | 13 | 122 | Focal | F | CBZ, CLB, LMT | TSC2 |
| T8 ^a^ | TSC | m | 6 | 14 | 122 | Focal | F | CBZ, VPA | TSC1 |
| T17 ^a^ | TSC | m | 0 | 5 | 91 | Focal | F | VPA, VGB | TSC2 |
| T20 ^a^ | TSC | f | 0 | 8 | 122 | Focal | F | CBZ, CLB | TSC2 |
| T21 ^a^ | TSC | f | 8 | 9 | 122 | Focal | F | CLB, OXC | TSC2 |
| T25 ^a^ | TSC | f | 0 | 17 | 22 | Focal | P | LEV, OXC, VPA, VGB | TSC2 |
| T27 ^a^ | TSC | f | 5 | 11 | 30 | Focal | F | CLB, TPM, VGB | TSC2 |

m = male, f = female, y = year, C = cortex, F = frontal, T = temporal, P = parietal, O = occipital, CBZ = Carbamazepine, CLB = Clobazam, CNP = Clonazepam, LCS = Lacosamide, LEV = Levetiracetam, LMT = Lamotrigine, OXC = Oxcarbazepine, PB = Phenobarbital, PER = Perampanel, PHT = Phenytoin, TPM =Topiramate, VGB = Vigabatrin, VPA = Valproic.acid, ZNS = Zonisamide, ^a^ = RNA sequencing
