# Supplementary material for: Down-regulation of the brain-specific cell-adhesion molecule contactin-3 in tuberous sclerosis complex during the early postnatal period
Source: J Neurodev Disord. 2022 Jan 15;14:8. doi: 10.1186/s11689-022-09416-2 (PMC8903535; doi:10.1186/s11689-022-09416-2)
Supplement: Supplementary file 2 — Additional file 2. [file 11689_2022_9416_MOESM2_ESM.pdf]

A

RNAseq TSC vs controls			
**Gene**	**Log2 (FC)**	**p-value**	**q-value**
CNTN1	-0.43	1.8E-01	7.5E-01
CNTN2	0.49	1.4E-01	6.9E-01
CNTN3	-1.32	5.0E-05	4.4E-03
CNTN4	-0.44	1.8E-01	7.6E-01
CNTN5	-0.45	1.4E-01	7.0E-01
CNTN6	0.08	8.4E-01	1.0E+00

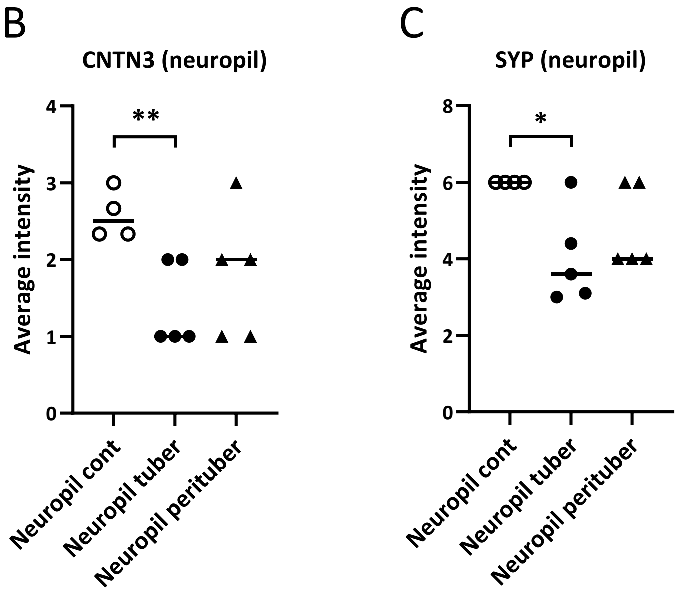

Supplementary Figure 1. (**A**) – RNAseq data indicating that *CNTN3* was the most significantly down-regulated gene of the contactin family (log2 fold change = -1.32, q-value = 0.004, n = 12 TSC vs n = 10 controls); (**B**) – Average immunoreactivity was lower in neuropil in cortical tubers compared to control; (**E**) – Average immunoreactivity for synaptophysin (SYP) was reduced in neuropil in cortical tubers; *p<0.01, Student's t-test
